# Thermal Oscillations of Nanobubbles

**DOI:** 10.1021/acs.nanolett.3c03052

**Published:** 2023-12-04

**Authors:** Duncan Dockar, Livio Gibelli, Matthew K. Borg

**Affiliations:** School of Engineering, Institute for Multiscale Thermofluids, University of Edinburgh, Edinburgh EH9 3FB, U.K.

**Keywords:** nanobubbles, cavitation, oscillations, nonequilibrium gas, nonideal gas, thermodynamics

## Abstract

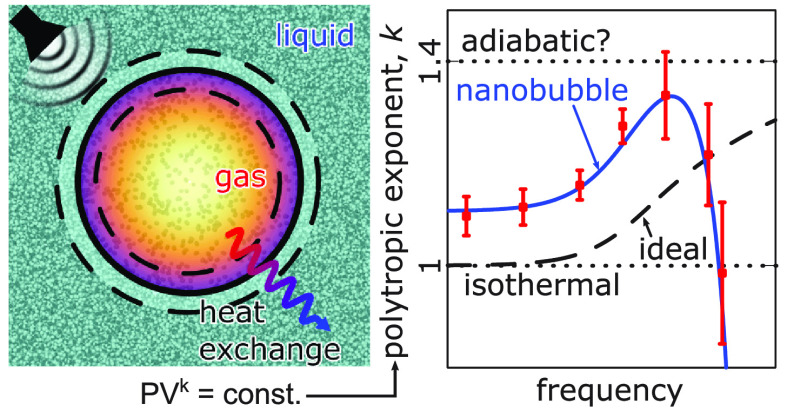

Nanobubble cavitation is advancing technologies in enhanced wastewater treatment, cancer therapy and diagnosis, and microfluidic cleaning. Current macroscale models predict that nanobubble oscillations should be isothermal, yet recent studies suggest that they are adiabatic with an associated increase in natural frequency, which becomes challenging when characterizing nanobubble sizes using ultrasound in experiments. We derive a new theoretical model that considers the nonideal nature of the nanobubble’s internal gas phase and nonequilibrium effects, by employing the van der Waals (vdW) equation of state and implementing a temperature jump term at the liquid–gas interface, respectively, finding excellent agreement with molecular dynamics (MD) simulations. Our results reveal how adiabatic behavior could be erroneously interpreted when analyzing the thermal response of the gas using the commonly employed polytropic process and explain instead how nanobubble oscillations are physically closer to their isothermal limit.

Cavitation is a complex fluid phenomenon, where bubbles can be induced to grow, oscillate, and collapse in response to external pressure fields. While the high-speed liquid jets that are released during their collapse are often considered destructive at the macroscale,^1^ cavitation has shown promise in breaking down and removing contaminant particles at the nanoscale,^2−6^ and nanobubbles are being proposed for enhanced disinfection in water and wastewater treatment,^4,7−11^ increased ultrasound imaging resolution,^12−14^ noninvasive and targeted drug delivery,^5,15−18^ and surface cleaning of microfluidic devices.^3^ Additionally, nanobubbles are particularly suited for traveling through microfluidic networks, such as extravascular spaces, the blood–brain barrier, and tumors, in much higher concentrations than larger bubbles (see Figure 1a–c),^13,17−20^ as well as a theorized diffusive stability that allows them to be utilized across much longer time scales than microbubbles.^21−23^ Cavitation bubbles are commonly described via the polytropic process,^1,24−29^ which not only governs their growth rate and natural frequency (similar to the stiffness of a harmonic oscillator)^26^ but also qualitatively describes the thermal behavior of the gas phase. Understanding this thermal/mechanical response has become crucial in experiments, where probing bubble resonant radii using ultrasonic frequencies is a common way of characterizing their mean size and distribution.^1,13,30,31^

**Figure 1:**
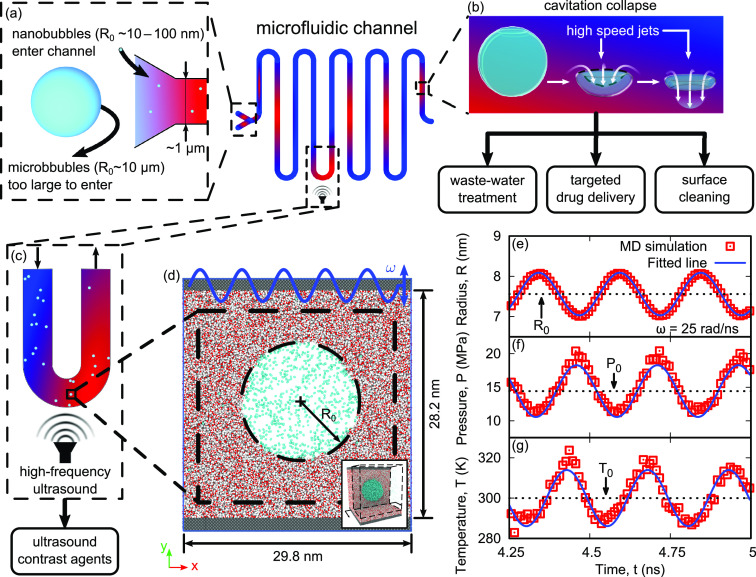
Schematic showing nanobubbles being employed in a microfluidic channel for cavitation applications. Insets show enhanced views of (a) nanobubbles entering microfluidic networks, that microbubbles are too large to reach, (b) the high-speed jets released during the final collapse stage, which have been proposed for the novel cavitation applications shown, and (c) nanobubbles being stimulated to oscillate using high-frequency ultrasound, such as in ultrasound contrast agents. (d) Molecular dynamics (MD) simulation setup for our nanobubble simulations, forced to oscillate using a vibrating piston, shown with a sliced view. The oxygen atoms are shown in red, hydrogen atoms in white, nitrogen atoms in cyan, and wall/piston atoms in gray. The inset shows an orthographic view of the three-dimensional domain, with some water molecules in the dashed box removed for clarity. Variation in (e) nanobubble radius *R*, (f) mean internal gas pressure *P*, and (g) mean internal gas temperature *T*, with time *t*, for the ω = 25 rad/ns oscillation case.

Early theoretical models assumed an ideal gas and uniform pressure within the bubble and proved that the transition from isothermal to adiabatic behavior is dependent on the Péclet number
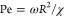
1where ω is the angular oscillation frequency, *R* is the bubble radius, and χ is the thermal diffusivity of the gas.^26−28^ The Péclet number is the ratio of advective transport to diffusive heat transport or, in this context, can be considered as a nondimensional oscillation frequency: i.e. at low Pe, the rate of heat exchange inside the bubble is sufficient to enable isothermal conditions during its oscillation, while at higher Pe, there is inadequate time for heat transfer, and the bubble interior tends to adiabatic behavior instead.^1,26−28^ More accurate formulations furthered this theory by considering nonuniform pressure distributions within the bubble and revealed an interrupted transition to adiabatic expansion, as a function of the Mach number Ma, before standing wave interference patterns instead dominate the radial pressure profile.^28,29,32,33^ Although suitable at the macroscale, these classical models fail to predict the apparent adiabatic oscillations that have been recently observed in surface nanobubbles when driven near their natural frequency.^25^

In this Letter, we investigate the thermal oscillations of nanobubbles and show how adiabatic behavior could be misinterpreted as a consequence of (a) nonideal gas behavior, due to the high Laplace pressure, and (b) nonequilibrium effects at the liquid–gas interface. We propose a new model that captures these nanoscale phenomena and show, with comparisons to molecular dynamics (MD) simulations, that nanobubble growth is in fact physically closer to the isothermal limit for a nonideal gas. For nanobubbles, the nonideal gas behavior is found in this work to be best described by the van der Waals (vdW) equation of state
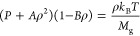
2where *P*, *T*, and ρ are the gas pressure, temperature, and density, respectively, *A* and *B* are empirically fitted constants, *M*_g_ is the mass of one gas molecule, and *k*_B_ is the Boltzmann constant. Additionally, the gas behavior departs from the continuum fluid assumption of local quasi-thermodynamic equilibrium and is found here to manifest as a Smoluchowski temperature jump at the liquid–gas interface, which is implemented as a boundary condition in our model^34−38^
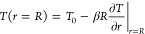
3where *T*_0_ is the equilibrium gas temperature and β is a dimensionless constant, given by
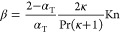
4where α_T_ is the thermal accommodation coefficient, which typically varies between 0 and 1 for fully specular and diffuse reflections, respectively. Pr is the Prandtl number, given by Pr  = μ_g_/ρχ, where μ_g_ is the gas viscosity, κ is the specific heat ratio of the gas, and Kn is the Knudsen number, given by , or the ratio between the mean free path and bubble radius.^37,38^ While eqs 3 and 4 were originally derived for a rarefied gas, we show in the Supporting Information (SI) that they provide a good estimate for the temperature jump in our high-density nonideal gas case, when modeling heat flux through a planar liquid–gas–liquid slab configuration, with α_T_ = 1. We take some crucial assumptions here by considering eq 3 as a boundary condition. First, we assume that the internal gas phase is noncondensable, which holds for a relatively insoluble gas such as nitrogen, where the time scale for diffusive growth of ∼1 μs^21,39^ is much larger than the oscillation period at the nanobubble’s natural frequency ∼0.1 ns,^1,25,28,29^ and in the SI, we show negligible mass transfer in our MD simulations. Second, the liquid temperature is assumed to be constant during nanobubble oscillations, which is suitable when the thermal conductivity of the liquid is much larger than that of the gas.^27,29^ Finally, eq 3 is a first-order jump condition, which is appropriate in the *slip flow* regime (10^–2^ < Kn < 0.1),^34,35,40,41^ where nonequilibrium behavior can be assumed to be relevant only in a thin region next to the liquid–gas interface—the Knudsen layer—while the interior bulk of the bubble is in local quasi-thermodynamic equilibrium.^42^

Using eqs 2 and 3, we derive an analytical model in the SI, which describes the nanobubble’s internal pressure and temperature variations when undergoing linear radial oscillations, where ϕ, θ, and ξ (≪ 1) are the respective nondimensional perturbations, e.g. *P* = *P*_0_(1 + ϕ) and *T* = *T*_0_(1 + θ), with the 0 subscripts denoting equilibrium values. The setup of our MD simulations consists of a nitrogen nanobubble in water with equilibrium radius *R*_0_ = 7.56 nm, being subjected to pressure waves using a piston,^6,24,25,43^ oscillating at various frequencies ω = 0.5–125 rad/ns, as shown in Figure 1d.^1^ For each case, the nanobubble radius, mean gas pressure, and temperature exhibited sinusoidal oscillations, matching the input piston frequency, as shown in Figure 1e–g, respectively, for the ω = 25 rad/ns (Pe = 8.5) case.

**Figure 2:**
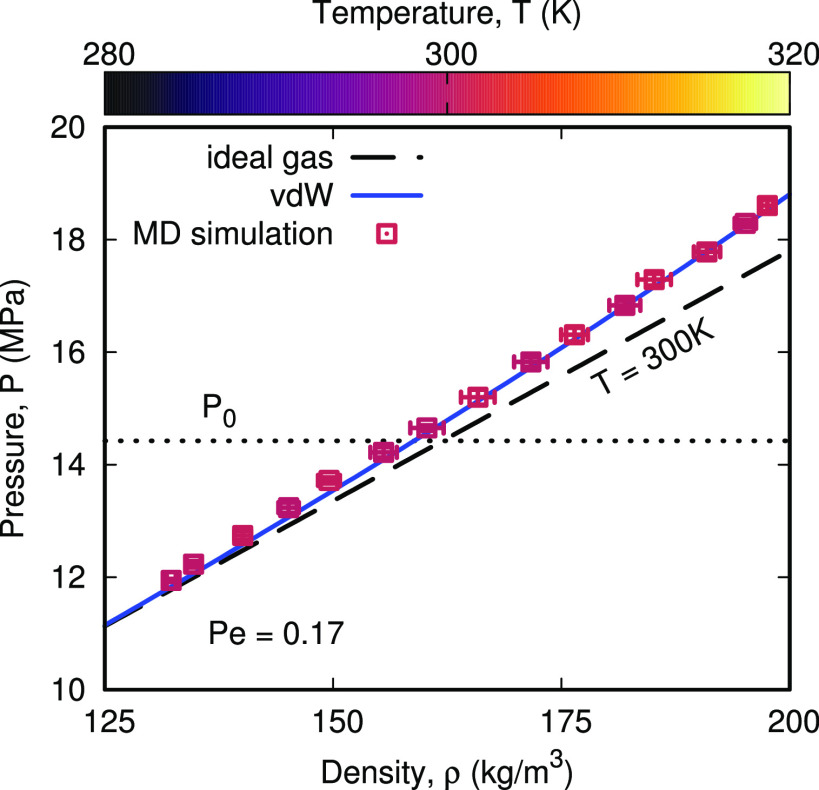
Variation in the nanobubble’s gas pressure *P* (and temperature *T*, shown in color) with density ρ, for the Pe = 0.17 oscillation case. MD simulation results are compared with the vdW equation of state in eq 2, and the ideal gas law *P* = ρ*k*_B_*T*/*M*_g_, both at *T* = 300 K. The equilibrium gas pressure *P*_0_ is also shown as a dotted line.

Our simulations confirm the presence of vdW gas behavior, as shown in Figure 2, for the slowest case Pe = 0.17. Temperature is shown by color, which is roughly constant within the bubble (when compared to the variation in Figure 1g), as expected at the limit of low Pe, and the MD results are well predicted by eq 2 for isothermal conditions at *T* = 300 K, but not the ideal gas law *P* = ρ*k*_B_*T*/*M*_g_. The effects of the interfacial temperature jump are evident when we look at the local pressure and temperature inside the gas for a higher Pe case. Following from our derivation, which now includes eqs 2 and 3, we can split the profiles into radial *r* and temporal *t* components, e.g. ϕ(*r*,*t*) = −ξ(*t*)ϕ̅(*r*), where both the pressure ϕ̅ and temperature θ̅ amplitudes take hyperbolic forms (see the SI), as shown in Figures 3a,b, respectively, for the Pe = 17 case. Further examples for other values of Pe are given in the SI.

**Figure 3:**
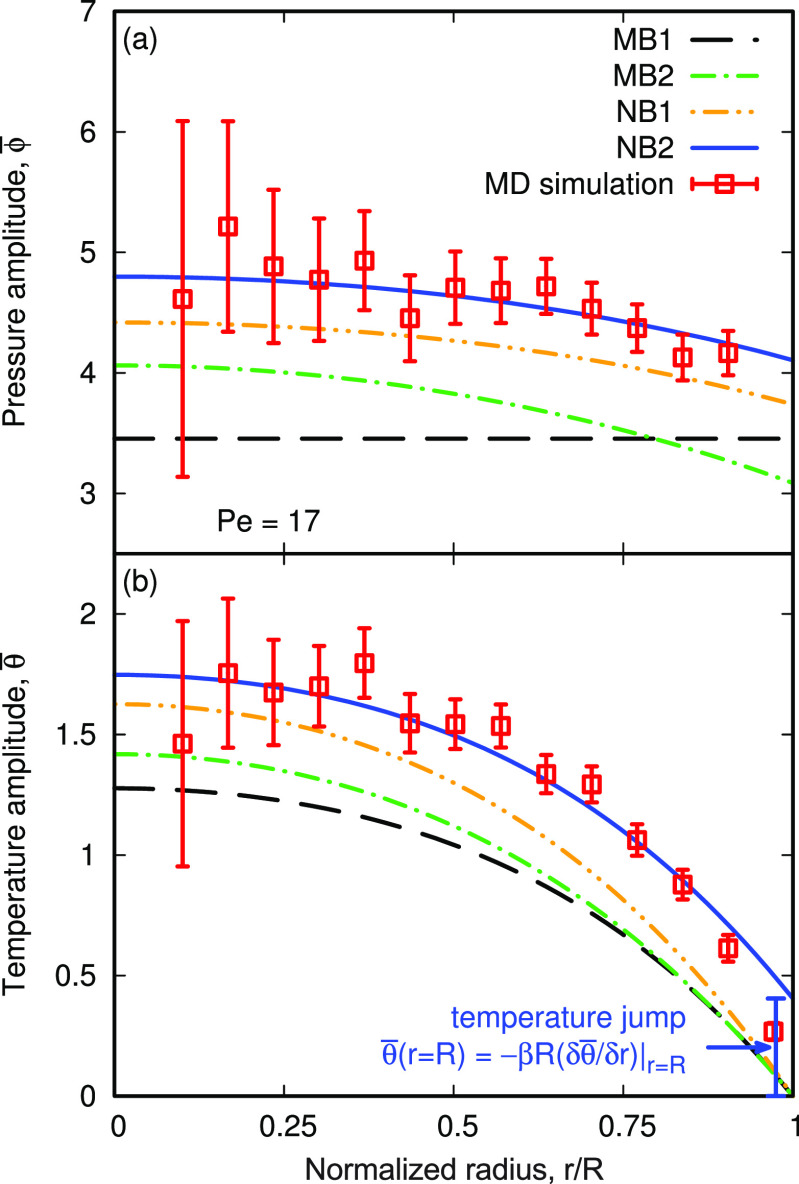
Radial variations of (a) pressure ϕ̅ and (b) temperature θ̅, nondimensional amplitudes in the oscillating nanobubble, for the Pe = 17 case. The temperature jump θ̅(*r* = *R*) at the liquid–gas interface is shown. The model cases, and their key assumptions, are summarized in Table 1.

In order to evaluate the limitations of the classical bubble theories, we first compare our MD results to two benchmark macroscale bubble models, employing similar assumptions to those found in refs (27−29, 32, and 33). To parametrize the different gas effects, we define linearized nonideal coefficients *W* = (ρ/*P*)(*∂P*/*∂ρ*)_T_ and *J* = (*T*/*P*)(*∂P*/*∂T*)_ρ_, obtained from eq 2, which both tend to unity for an ideal gas. Compressibility effects are captured using a proposed nondimensional number *D* = χ^2^ρ/(*PR*^2^), which is related to the Mach number by Ma ≈ Pe(*D*/κ)^1/2^,^28^ while the nonequilibrium temperature jump is characterized by β in eq 4. The various coefficients for the different analytical models investigated here are summarized in Table 1 and apply to all the following figures where used.

**Table 1:** Summary of the Different Macrobubble (MB) and Nanobubble (NB) Model Cases Considered Here, Their Key Assumptions, and the Corresponding Coefficients Used in Our Modeling

case	assumptions	coefficients
MB1	ideal gas	*W* = *J* = 1
	uniform pressure distribution	*D* → 0
	no temperature jump at interface	β = 0
		
MB2	ideal gas	*W* = *J* = 1
	nonuniform pressure distribution	*D* = 7.2 × 10^–3^
	no temperature jump at interface	β = 0
		
NB1	vdW gas	*W* = 1.11, *J* = 1.27
	nonuniform pressure distribution	*D* = 5.5 × 10^–3^
	no temperature jump at interface	β = 0
		
NB2	vdW gas	*W* = 1.11, *J* = 1.27
	nonuniform pressure distribution	*D* = 5.5 × 10^–3^
	temperature jump at interface	β = 0.12

The first benchmark macrobubble model (MB1) assumes an ideal gas and low Mach number such that the internal pressure is uniform throughout the bubble and there is no temperature jump at the liquid–gas interface, which can be equivalently obtained by setting *W* = *J* = 1, *D* → 0, and β = 0. However, our MD simulations in Figure 3a show that pressure is not uniform, and therefore, MB1 is unlikely to accurately predict the nanobubble’s oscillation behavior. Alternatively, MB2 assumes an ideal gas and finite Mach number (by setting *D* = 7.2 × 10^–3^), which correctly identifies a nonuniform pressure, although still underpredicts the MD results. We introduce our new nanobubble (NB) models, by initially considering a vdW gas (without a temperature jump) by setting *W* = 1.11, *J* = 1.27, and *D* = 5.5 × 10^–3^ in NB1,^2^ where we see a small improvement. However, it is only when we also include the temperature jump in model NB2 with β = 0.12 (calculated using eq 4, with α_T_ = 1) that we see the best agreement with our MD simulations. Similarly, MB1 and MB2 underpredict temperature in Figure 3b; however, we find much better agreement with our proposed models when considering the vdW gas and crucially the temperature jump at the liquid–gas interface θ̅(*R*) > 0, which is only captured by NB2.

After confirming that our equations accurately predict the internal gas phase’s thermodynamic behavior, we now look to evaluate the pressure at the liquid–gas interface, such that we can express the nanobubble’s expansion equivalently to the polytropic gas law *PR*^3*k*^ = const, which is necessary to estimate gas pressure in inertial cavitation models, such as the Rayleigh–Plesset equation.^1,24,25,27−29,44,45^ Linearizing the polytropic gas law gives
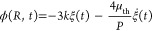
5where dot notation denotes time derivatives, e.g. ξ̇ = dξ/d*t*.^1,26−29^ The polytropic exponent *k* is commonly understood to vary between 1 and κ_i_, for isothermal and adiabatic gas expansion, respectively, where κ_i_ is the specific heat ratio for an ideal gas, and equal to 1.4 when considering diatomic molecules.^1,24−30,32,33^ Alongside *k* is a corresponding thermal viscosity μ_th_ term, which accounts for the energy losses due to the thermal conduction between the gas and liquid phases and can be summed with the liquid dynamic viscosity μ for estimating the equivalent total bubble damping.^1,26−29^ Determining the thermal viscosity is crucial in applications where inaccurate estimation of the damping can yield lower than expected oscillation amplitudes, such as in ultrasound contrast agents.^12,13^ The polytropic exponent and thermal viscosity can then be found by evaluating the pressure amplitude at the interface:
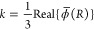
6and
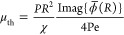
7

Figure 4a,b shows the variation in polytropic exponent *k* and nondimensional thermal viscosity μ_th_/(*PR*^2^/χ), respectively, with Péclet number Pe. Our MD results (fitted from eq 5) show an increase in *k* with Pe before reaching a maximum of 1.33 at Pe ≈ 17 (or Ma ≈ 1).^29^ Neither the MB1 nor MB2 model accurately predicts this behavior; in fact, MB2 suggests that nanobubbles should never achieve adiabatic expansion and would always appear roughly isothermal (*k* ≈ 1) for Pe < 20. Instead, our NB2 model completely captures this variation. For increasing frequencies, standing waves develop within the bubble that dominate the pressure profile, resulting in repeating peak and trough patterns in *k*.^29^ We deliberately limited our MD simulations to Péclet numbers below this regime, since taking accurate measurements with these interference patterns is challenging.

From our MD results, it would appear that adiabatic oscillations of nanobubbles are reasonable, since *k* ≈ κ_i_ at its maximum, similar to recent observations.^25^ However, at low Péclet numbers (Pe ≪ 1), we do not observe the expected limit of *k* = 1 for isothermal expansion in Figure 4a, despite us finding constant bubble temperatures for the slowest Pe = 0.17 case in Figure 2. Instead, the vdW gas properties have shifted the limits of *k* for isothermal and adiabatic behavior to *W* ≈ 1.11 and κ*W* ≈ 1.77, respectively,^46^ where κ is the specific heat ratio of the vdW gas (see Figure 4a). Note that since the gas is described by eq 2, common thermal properties are no longer equal to their ideal counterparts, e.g. κ ≠ κ_i_.^47^ Now it becomes apparent that the nanobubble oscillations are not physically adiabatic despite sharing a similar polytropic exponent with an adiabatic ideal gas. Qualitatively, the measured polytropic exponents are nearer their vdW isothermal limit *W*, although the predicted values of *k* in Figure 4a are still quantitatively useful in estimating the nanobubble’s natural frequency (as we do later). We emphasize that the nonequilibrium temperature jump does partially insulate the bubble and still promotes genuine adiabatic behavior at higher Pe, over and above the revised vdW isothermal–adiabatic limits, as highlighted by the difference between the NB1 and NB2 models in Figure 4a. So far, we have only focused on the polytropic exponent of the gas; however, the thermal viscosity is also strongly affected by both vdW and nonequilibrium effects, with NB2 reaching over twice the magnitude as that expected by the classical bubble models for Pe ≪ 1, as shown in Figure 4b. Our model assumes an inviscid gas phase, which is valid for ω ≪ *P*/μ_g_ (approximately ω ≪ 640 rad/ns, or Pe ≪ 220 for our case). For simplicity, we have also neglected the *acoustic viscosity* in our modeling, another damping mechanism which is equivalent to dissipation from sound wave radiation within the (compressible) liquid phase, since these effects only become relevant for high liquid Mach numbers Ma_l_ = ω*R*/*c*_l_ ≈ 1 (ω ≈ 200 rad/ns in our case), where *c*_l_ is the liquid sound speed.^1,26−28,48^ However, this could explain the small discrepancies in our MD μ_th_ measurements for the higher frequency cases at Pe ≈ 40. The error bars in MD thermal viscosity for Pe < 1 are large, since the thermal noise and low frequency make it difficult to accurately fit for the ξ̇ (radial velocity) term in eq 5.

We have demonstrated that our model accurately describes the thermal oscillation behavior of a *R* ≈ 10 nm nanobubble in our MD simulations, but what does it predict for a broader range of bubble sizes? Using more realistic experimental parameters for a nitrogen bubble in water (i.e., surface tension γ and vdW parameters *A* and *B*)^47−49^ we plot the variations in β, *D*, *W*, and κ*W* in Figure 5a and the resulting variations in *k* and thermal viscosity μ_th_ in Figure 5b,c, respectively, when evaluated at their natural frequency ,^1^ where ρ_*l*_ is the liquid density. We find β and *D* are roughly constant and at their maximum values for *R* < 10^–6^ m, since the Laplace pressure dominates the internal pressure, i.e. *P* ≈ 2γ/*R*, suggesting the effects of the nonequilibrium gas and nonuniform pressure are similar for nearly all nanobubble sizes.^42^ Conversely, the gas becomes more nonideal as the Laplace pressure increases, so *W* and κ deviate from their ideal values in this nanobubble range, and we see further changes in the effective polytropic exponent and thermal viscosity, in Figure 5b,c, respectively. For the *R* = 10 nm nanobubble, *k* reaches around 1.25, although as discussed previously, this does not necessarily indicate more adiabatic expansion, since the limits of isothermal and adiabatic behavior have also increased at this scale, as shown in the inset in Figure 5a. We also find that *W* decreases below unity for some nanobubble sizes *R* ≈ 30 nm, which would suggest other nontrivial polytropic behavior is possible: e.g., isothermal behavior at *k* < 1. Thermal viscosity in our NB2 model remains significantly larger (∼50%) than that predicted by the classical models, reaching a maximum at *R* ≈ 100 nm.

**Figure 4:**
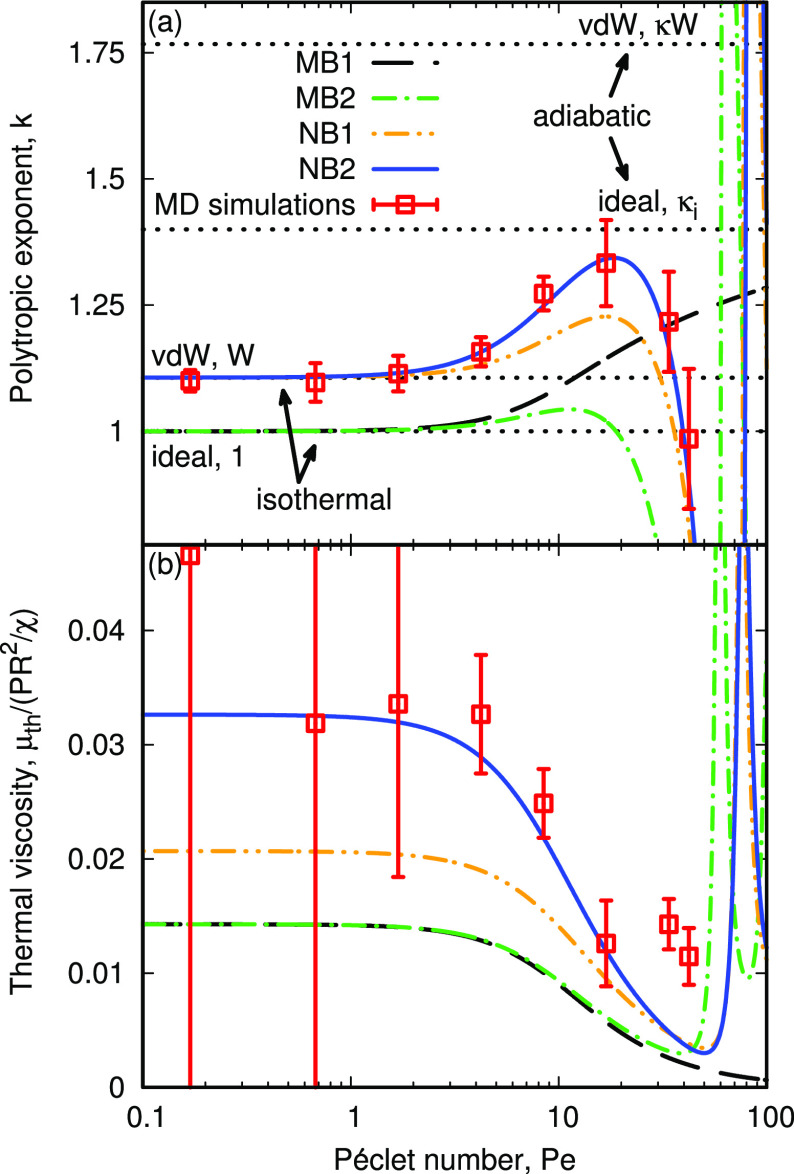
Variation in (a) polytropic exponent *k* and (b) nondimensional thermal viscosity μ_th_/(*PR*^2^/χ) with Péclet number Pe. The dotted lines in (a) show the limits for isothermal and adiabatic expansion for an ideal gas, *k* = 1 and *k* = κ_*i*_ = 1.4, respectively, and the vdW gas, *k* = *W* ≈ 1.11 and *k* = κ*W* ≈ 1.77, respectively. The model cases, and their key assumptions, are summarized in Table 1.

**Figure 5:**
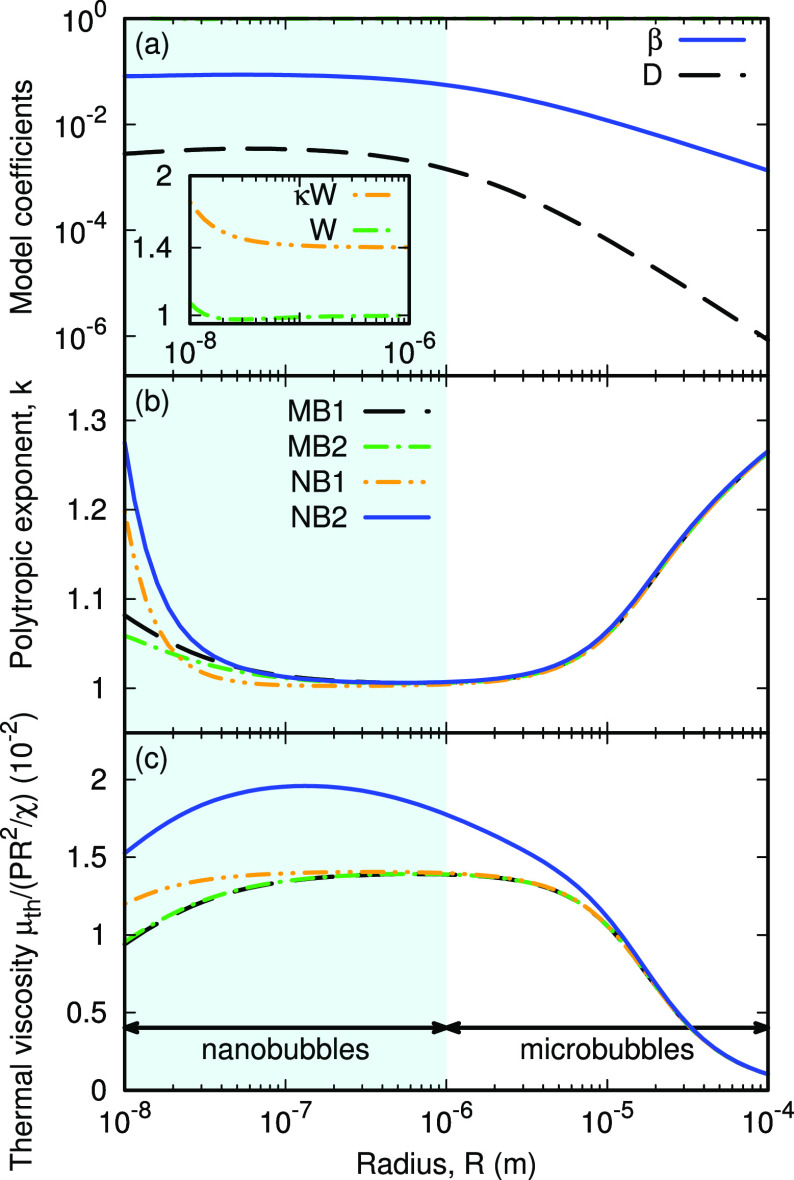
Variation in (a) coefficients β, *D*, and (inset) *W* and *κW*, representing the polytropic limits for isothermal and adiabatic expansion, respectively, (b) polytropic exponent *k*, and (c) thermal viscosity μ_th_ (normalized by *PR*^2^/χ), with equilibrium radius *R*, when evaluated at the bubble’s natural frequency ω_n_. The legend in (b) also applies to (c). The model cases, and their key assumptions, are summarized in Table 1.

In summary, we have demonstrated how nanobubble thermal oscillations can only be accurately captured by considering the gas’ (a) nonideal properties, which we model with the vdW equation of state, and (b) nonequilibrium behavior, which manifests as a temperature jump at the liquid–gas interface. Our Letter shows compelling evidence that the existence of adiabatic oscillations in nanobubbles reported recently is likely not physical.^25^ The higher polytropic values observed need to be considered in the reference frame for more physical isothermal and adiabatic limits appropriate for nanobubbles. In another of our previous works, in which we investigated the growth and cavitation threshold of surface nanobubbles, we fitted a polytropic value of *k* = 1.18, which we now conclude was expanding at its nonideal isothermal limit *W*.^24^ Future work on this topic could further explore nonequilibrium behavior with complete kinetic modeling of the internal gas phase, particularly in the special case of nanobubbles stabilized by reduced surface tension organic shells,^13,14,17−20,42,50−54^ where higher Knudsen numbers Kn ≳ 0.1 would elicit stronger nonequilibrium effects. Mass transfer could also be incorporated into our model, to capture the evaporation and condensation of gas/vapor molecules, e.g. in plasmonic nanobubbles,^30,55−57^ and the nonideal and nonequilibrium behavior may explain other nontrivial adiabatic behavior observed, e.g. *k* > κ_*i*_ in ref (56).

## Supporting Information Available

Description of our MD simulation setup, derivation for our nanobubble thermal oscillation model, nonequilibrium MD simulations of the temperature jump at the liquid–gas interface, equilibrium MD simulations and fitting to the vdW equation of state, and derivation of the updated isothermal and adiabatic polytropic limits for a vdW gas (PDF)
