# Supplementary material for: Thermal Oscillations of Nanobubbles
Source: Nano Lett. 2023 Dec 4;23(23):10841–7. doi: 10.1021/acs.nanolett.3c03052 (PMC10722608; doi:10.1021/acs.nanolett.3c03052)
Supplement: Supplementary file 1 — nl3c03052_si_001.pdf [file nl3c03052_si_001.pdf]

Supporting Information: Thermal oscillations of nanobubbles

Duncan Dockar,* Livio Gibelli, and Matthew K. Borg

School of Engineering, Institute for Multiscale Thermofluids, University of Edinburgh, Edinburgh EH9 3FB, UK

E-mail: d.dockar@ed.ac.uk

Contents

S1 Molecular Dynamics simulation **S-2**

S2 Derivation of nanobubble thermal oscillation model **S-4**

S2.1 Evidence for non-condensable gas in nanobubble S-12

S2.2 Pressure and temperature variations in nanobubble S-14

S3 Van der Waals equation of state **S-14**

S4 Molecular Dynamics simulations of Knudsen temperature jump **S-18**

S5 Van der Waals polytropic gas law limits **S-20**

S1 Molecular Dynamics simulation

We used the Large-scale Atomic/Molecular Massively Parallel Simulator (LAMMPS) software for all Molecular Dynamics (MD) simulations.[S1] All interatomic interactions were modeled by the Lennard–Jones (LJ) and Coulomb potentials:

$$U_{ij}(r_{ij}) = 4\epsilon_{ij}\left[\left(\frac{\sigma_{ij}}{r_{ij}}\right)^{12} - \left(\frac{\sigma_{ij}}{r_{ij}}\right)^{6}\right] + \frac{1}{4\pi\epsilon_0}\frac{q_i q_j}{r_{ij}}, \tag{S1}$$

where ϵ_{ij} and σ_{ij} are the LJ potential well-depth and characteristic length-scale, respectively, between atoms i and j, separated by a distance r_{ij}; q_i and q_j are the charges of atoms i and j, respectively, interacting via the Coulomb potential, and ϵ_0 is the permittivity of free-space.[S1] To reduce computational costs, potentials were truncated and shifted, with cut-off radii (r_c) of 1.65 nm and 1.45 nm for the LJ and Coulombic potentials, respectively.[S1,S2]

We placed 8×10^6 TIP4P/2005 water (H_2O) molecules for the liquid phase, each comprising two hydrogen (H) atoms, one oxygen (O) atom, and a charged massless particle (M), in between two parallel Face Centered Cubic (FCC) walls, composed of wall (W) atoms. A gas nanobubble was initialized as a sphere, containing 7308 two-site nitrogen (N_2) molecules, with a further 1281 N_2 molecules dissolved in the surrounding liquid to achieve supersaturation.[S3,S4]

Interatomic potential parameters and masses for each atom type are given in Table S1. Fixed boundaries were applied in the y direction, and periodic boundary conditions were applied in the x and z directions. Simulations were time-integrated using the velocity Verlet algorithm,[S1] with a time-step of $\Delta t = 1$ fs. During 2 ns of equilibration, Nosé–Hoover thermostats at 300 K were applied to the H_2O and N_2 molecules, while a constant pressure equivalent to $P_{\infty,0} = 0.1$ MPa was applied to the top wall, acting as a piston.[S2,S6,S7] To prevent net motion of the system, the lower wall was fixed in position. The equilibrated domain had dimensions $29.8 \times 28.2 \times 29.8\,\text{nm}^3$, although was allowed to vary in the y direction to

Table S1: Atom types and parameters for interatomic potential interactions.[S4–S6] LJ parameters for atoms in bold are given for pairs of like atoms. Any interaction pairs not given are equal to zero.

**Atom**/ Interatomic pair	Atom Mass (g/mol)	ϵ (kJ/mol)	σ (nm)	q (e)
**H**	1.008	0	0	0.5564
**M**	0	0	0	−1.1128
**O**	15.9994	0.7749	0.3159	0
O–N	-	0.5456	0.3243	-
O–W	-	1.3548	0.2815	-
**N**	14.007	0.3026	0.3320	0
N–W	-	0.8422	0.2815	-
**W**	15.9994	66.9607	0.2471	0

accommodate the piston motion.

We determined the liquid-gas interface by the 50 % isodensity contour, and fitted a spherical profile, to measure the equilibrated bubble radius as $R_0 = (7.56 \pm 0.02)$nm, and internal gas density $\rho_0 = (159 \pm 2)\text{kg/m}^3$. Atomic positions, velocities, and forces were regularly outputted, to measure the variations in nanobubble radius, pressure, and temperature. The unbiased pressure was measured by subtracting the mean flow velocity, which was necessary for the high oscillation frequency cases during the main production runs, and was measured locally within concentric spherical shelled-bins inside the nanobubble, using the virial theorem:[S1,S2,S6,S8,S9]

$$P = \frac{1}{3V_{bin}} \left[\sum_j M_j \left| \vec{u}_j - \langle \vec{u} \rangle \right|^2 + \frac{1}{2} \sum_j \sum_{k \neq j} \vec{F}_{jk} \cdot \vec{x}_{jk} \right], \tag{S2}$$

where V_{bin} is the bin volume, M_j is the mass of atom j in the bin, u_j is the atomic velocity, $\langle \vec{u} \rangle = \sum_j (M_j \vec{u}_j) / \sum_j M_j$ is the mean flow velocity in the bin, and $\vec{F}_{jk}$ and $\vec{x}_{jk}$ are the force and separation vectors acting between atoms j and k. The initial gas pressure was measured $P_0 = (14.4 \pm 0.3)$MPa, and was used to estimate the surface tension γ from the Laplace pressure at equilibrium conditions: $\gamma = (P_0 - P_{\infty,0})R_0/2 = (54 \pm 1)\text{mJ/m}^2$, which is lower than the experimental value of water, since we have used truncated and shifted

LJ and Coulombic potentials in Equation (S1).[S10] Including long-range interactions of these potentials is not trivial to compute surface tension, due to the highly non-isotropic and non-homogeneous multiphase fluid setup in our simulations,[S11–S13] however, is also not necessary for the purposes of our work.[S2,S6]

Similarly, unbiased temperature was measured locally from:

$$T = \sum_j \frac{M_j}{N k_B} \left| \vec{u}_j - \langle \vec{u} \rangle \right|^2, \tag{S3}$$

where N is the number of degrees of freedom for atom j (see Section S2 below).

During the main production runs, the top piston forcing was removed, and instead was set to oscillate sinusoidally at a given frequency $\omega = 0.5$–$125\,\mathrm{rad/ns}$ with a fixed amplitude between 0.08–0.45 nm, for up to 5 ns (except for the 0.5 ns case, which was run for 24 ns to obtain at least one full oscillation cycle). This method was found to best enforce stable linear oscillations of the nanobubbles with constant amplitude, rather than through imposing a sinusoidal forcing in previous works,[S2,S6] which was subject to non-linearities from the piston mass and thermal noise. We decreased the oscillation amplitude for increasing frequencies to ensure bubble oscillations remained linear and to prevent excess liquid heating.[S14,S15] The top and bottom wall atoms were thermostated to 300 K, using a Berendsen thermostat, applied only to the x and z thermal velocity components.

S2 Derivation of nanobubble thermal oscillation model

In this section, we derive the effective gas polytropic exponent k and thermal viscosity μ_{th} of a spherical nanobubble, initially at thermal and mechanical equilibrium, undergoing linear oscillations with frequency ω (see Equations (6) and (7) in the main text). We account for the non-ideal behavior of the internal gas phase using the van der Waals (vdW) equation of state, and non-equilibrium effects as a temperature jump at the liquid-gas interface (see Equation (3) of the main text), with further details for each assumption given in Sections S3

and S4, respectively.

We begin with the vdW equation of state, which is particularly suited for dense gases:[S16,S17]

$$\left(P + A\rho^2\right)(1 - B\rho) = \frac{\rho k_B T}{M_g}, \tag{S4}$$

where ρ is the mass-density of the gas,[1] k_B is the Boltzmann constant, M_g is the mass of one gas molecule, and A and B are fitted constants. Details of the fitting parameters of our two-site N_2 model with the vdW equation of state are given in Section S3.

We linearize the variations in pressure, density and temperature, such that they can be expressed as:

$$P(r,t) = P_0\left(1 + \phi(r,t)\right), \tag{S5a}$$

$$\rho(r,t) = \rho_0\left(1 + \eta(r,t)\right), \tag{S5b}$$

$$T(r,t) = T_0\left(1 + \theta(r,t)\right), \tag{S5c}$$

respectively, where $\phi(r,t)$, $\eta(r,t)$, and $\theta(r,t)$ are the non-dimensional variations in pressure, density and temperature, respectively, as functions of radial distance from the bubble centre r and time t. The 0 subscripts in P, ρ, and T indicate the equilibrium values. Similarly, the linearized perturbation in the radius R is given as:

$$R = R_0\left(1 + \xi(t)\right), \tag{S6}$$

where R_0 is the bubble's equilibrium radius, and $\xi(t)$ is the dimensionless radius perturbation. We will assume steady-state oscillations of the bubble,[2] such that the variables ϕ, η, θ, and ξ are small ($\ll 1$), and can be expressed as sinusoidal functions of time, e.g. for radius

[1] The vdW equation of state is conventionally expressed in terms of molar volume V_m, however, here we express it in terms of mass-density ρ, for easier comparisons with simulation measurements.[S16]

[2] Our assumption for steady-state oscillations is suitable for "short" ($\sim 5\,\text{ns}$) timescales, as in our simulations, where the mass of the bubble can be considered constant. For longer times, i.e. approaching the diffusive timescale ($\sim 1\,\mu\text{s}$ for a $R_0 \sim 10\,\text{nm}$ nanobubble)[S18] we would expect gradual growth under ultrasound irradiation, via *rectified diffusion.*[S3,S19]

$\xi(t) = \text{Real}\{\bar{\xi} \exp(i\omega t)\}$, where $\bar{\xi}$ is the non-dimensional radius oscillation amplitude.

Cavitation models typically assume a polytropic process for the gas phase, i.e. $PR^{3k} =$ const., where k is the polytropic exponent, so the gas pressure inside a bubble takes the following form:[S20,S21]

$$P = P_0 \left(\frac{R_0}{R}\right)^{3k} - \frac{4\mu_{th}\dot{R}}{R}. \tag{S7}$$

We also include a corresponding thermal viscosity term μ_{th}, which accounts for the equivalent energy dissipation during repeated heating and cooling cycles of the bubble oscillation,[S21–S24] and can be summed with the liquid dynamic viscosity μ in other cavitation models, e.g. the Rayleigh–Plesset equation.[S25,S26] Dot notation is used to indicate time derivatives, e.g. $\dot{R} = \mathrm{d}R/\mathrm{d}t$. Equation (S7) is linearized to give the expression:

$$\phi = -3k\xi - \frac{4\mu_{th}}{P_0}\dot{\xi}. \tag{S8}$$

We shall derive a model that relates the pressure to the radius perturbations, in a similar form to Equation (S8).

The equations in (S5) can be related by:

$$\phi = W\eta + J\theta, \tag{S9}$$

where $W = (\rho_0/P_0)(\partial P/\partial\rho)_T$ and $J = (T_0/P_0)(\partial P/\partial T)_\rho$. Using Equation (S4), the coefficients W and J can be more explicitly shown as:

$$W = \frac{\rho_0}{P_0}\left[\frac{k_B T_0}{M_g\left(1 - B\rho_0\right)^2} - 2A\rho_0\right], \tag{S10}$$

and

$$J = \frac{T_0}{P_0}\left[\frac{\rho_0 k_B}{M_g\left(1 - B\rho_0\right)}\right]. \tag{S11}$$

For an ideal gas, W and J are equal to unity, however, now using Equations (S10) and

(S11), we can obtain thermodynamic quantities for the vdW gas. For example, the specific heat capacity at constant volume c_V in the gas is expressed analytically as:

$$c_V = \frac{N_D k_B}{2M_g}, \tag{S12}$$

where N_D is the number of degrees of freedom per molecule; for a diatomic gas, such as nitrogen, $N_D = 5$. Using Maxwell's relations, we can find the specific heat capacity at constant pressure:

$$c_P = c_V + \frac{T_0 \zeta^2}{\rho_0 \psi}, \tag{S13}$$

where $\psi = (\partial \rho / \partial P)_T / \rho_0 \approx (W P_0)^{-1}$ is the isothermal compressibility, and $\zeta = -(\partial \rho / \partial T)_P / \rho_0$ $\approx J(W T_0)^{-1}$ is the volume expansion coefficient.[S16,S17] Using Equations (S12) and (S13), the specific heat capacity ratio $\kappa = c_P / c_V$ can therefore be expressed as:

$$\kappa = 1 + \frac{2M_g J^2 P_0}{N_D \rho_0 k_B T_0 W}. \tag{S14}$$

We shall derive a model that relates the pressure to the radius perturbations in a similar form to Equation (S8), by linearizing the Navier–Stokes–Fourier equations and assuming spherical symmetry. The conservation of mass reads:

$$\frac{\mathrm{D}\rho}{\mathrm{D}t} = -\rho \nabla \cdot \mathbf{u}, \tag{S15}$$

where $\mathbf{u}$ is the velocity vector in the gas; the operator $\mathrm{D}/\mathrm{D}t$ represents the material derivative, e.g. $\mathrm{D}\rho/\mathrm{D}t = \partial \rho / \partial t + \mathbf{u} \cdot \nabla \rho$. With spherical symmetry (i.e. by only considering the radial component r), Equation (S15) simplifies to:

$$\frac{\partial \rho}{\partial t} + u \frac{\partial \rho}{\partial r} = -\rho \frac{1}{r^2} \frac{\partial \left(u r^2\right)}{\partial r}, \tag{S16}$$

where u is the radial component velocity. Using Equation (S5), linearizing Equation (S16)

gives:

$$i\omega\eta = -\frac{1}{r^2}\frac{\partial\left(ur^2\right)}{\partial r}. \tag{S17}$$

Note that the operator $\partial/\partial t$ becomes equal to $i\omega$, for steady-state oscillations.[S21]

Conservation of momentum is:

$$\frac{\mathrm{D}(\rho\mathbf{u})}{\mathrm{D}t} = -\nabla P + \nabla\cdot\left[2\mu_g\left\{\frac{1}{2}\left(\nabla\mathbf{u} + \{\nabla\mathbf{u}\}^\top\right) - \frac{1}{3}\bar{\mathbf{I}}\nabla\cdot\mathbf{u}\right\}\right], \tag{S18}$$

which simplifies to:

$$\frac{\partial(\rho u)}{\partial t} + u\frac{\partial\left(\rho u\right)}{\partial r} = -\frac{\partial P}{\partial r} + \frac{4}{3}\mu_g\frac{\partial}{\partial r}\left(\frac{1}{r^2}\frac{\partial\left(r^2 u\right)}{\partial r}\right), \tag{S19}$$

assuming spherical symmetry, and where μ_g is the gas viscosity. Linearizing Equation (S19), making use of the identity in Equation (S17), we obtain:

$$i\omega u = -\frac{P_0}{\rho_0}\frac{\partial\phi}{\partial r} - \frac{4i\omega\mu_g}{3\rho_0}\frac{\partial\eta}{\partial r}. \tag{S20}$$

Here, we choose to neglect viscosity in the bulk gas, which is valid in the limit $\omega\mu_g/P_0 \ll 1$.

Finally, the energy equation for a gas, neglecting additional heat source terms, is:

$$\rho c_P\frac{\mathrm{D}T}{\mathrm{D}t} = \nabla\cdot(K\nabla T) + \zeta T\frac{\mathrm{D}P}{\mathrm{D}t} + \nabla\cdot\left(\left[2\mu_g\left\{\frac{1}{2}\left(\nabla\mathbf{u} + \{\nabla\mathbf{u}\}^\top\right) - \frac{1}{3}\bar{\mathbf{I}}\nabla\cdot\mathbf{u}\right\}\right]\cdot\mathbf{u}\right), \tag{S21}$$

where K is the thermal conductivity, predicted from:[S27,S28]

$$K = \mu_g\frac{k_B}{2M_g}\left(\frac{15}{2} + (N_D - 3)\right), \tag{S22}$$

gas viscosity is:[S28,S29]

$$\mu_g = \frac{0.998}{\sigma^2}\sqrt{\frac{k_B M_g T_0}{\pi^3}}, \tag{S23}$$

and σ is the molecular diameter (see table S1) for nitrogen gas. Equation (S21) becomes:

$$\rho c_P \left(\frac{\partial T}{\partial t} + u\frac{\partial T}{\partial r}\right) = K\frac{1}{r^2}\frac{\partial}{\partial r}\left(r^2\frac{\partial T}{\partial r}\right) + \zeta T\left(\frac{\partial P}{\partial t} + u\frac{\partial P}{\partial r}\right) + 2\mu_g\left[u\frac{2}{r}\left(\frac{\partial u}{\partial r} - \frac{1}{3}\frac{1}{r^2}\frac{\partial\left(r^2u\right)}{\partial r}\right) + \frac{\partial}{\partial r}\left(u\frac{\partial u}{\partial r}\right) - \frac{1}{3}\frac{\partial}{\partial r}\left(u\frac{1}{r^2}\frac{\partial\left(r^2u\right)}{\partial r}\right)\right], \tag{S24}$$

assuming spherical symmetry, which after linearization and neglecting viscosity again, becomes:

$$i\omega\theta = \chi\frac{1}{r^2}\frac{\partial}{\partial r}\left(r^2\frac{\partial\theta}{\partial r}\right) + \zeta\frac{P_0}{\rho_0 c_P}i\omega\phi, \tag{S25}$$

where $\chi = K/\rho_0 c_P$ is the gas thermal diffusivity. The last coefficient on the right hand side of Equation (S25) can be expressed in terms of the specific heat ratio κ, using Equations (S12) and (S13):

$$\zeta\frac{P_0}{\rho_0 c_P} = \frac{1}{J}\frac{\kappa - 1}{\kappa}. \tag{S26}$$

We should note that κ is frequently utilized in cavitation literature for describing adiabatic behavior. This value is commonly assumed equal to 1.4 for diatomic gases (or 5/3 for monatomic gases), which is only true for an ideal gas; we will therefore refer to the ideal gas value as κ_i from here on to avoid confusion. Here, the actual ratio of the specific heat capacities is usually larger than that for the ideal gas case, i.e. $\kappa > \kappa_i$.

By combining Equations (S9), (S17), (S20), and (S25), we can rewrite the energy balance in terms of ϕ:

$$R_0^4\frac{\partial^4(r\phi)}{\partial r^4} + \left[\frac{Pe^2D}{W} - iPe\right]R_0^2\frac{\partial^2(r\phi)}{\partial r^2} - \frac{iPe^3D}{W\kappa}r\phi = 0, \tag{S27}$$

using the thermal Péclet number $Pe = \omega R_0^2/\chi$, representing the ratio of convective to diffusive thermal transport rates. We define another dimensionless number $D = \chi^2\rho_0/(P_0R_0^2)$, which is related to the Mach number by $Ma \sim Pe(D/\kappa)^{1/2}$.

The general solution to Equation (S27) has the form:

$$\phi = (-\xi)\frac{R_0}{r}\left[C_1\sinh\left(B_1\frac{r}{R_0}\right) + C_2\sinh\left(B_2\frac{r}{R_0}\right)\right], \tag{S28}$$

where we have already applied boundary conditions for continuous derivatives at the bubble center, i.e. $(\partial\phi/\partial r)|_{r=0}= 0$, and $(\partial\theta/\partial r)|_{r=0}= 0$. We also include an additional factor of $(-\xi)$, so that Equation (S28) is expressed in a similar form to the linearized polytropic equation in Equation (S8). Coefficients B_1 and B_2 are given by:[3]

$$B_{1,2} = \sqrt{\frac{Pe}{2W}\left\{(iW - PeD) \pm \left[(iW - PeD)^2 + \frac{4iPeDW}{\kappa}\right]^{\frac{1}{2}}\right\}}. \tag{S29}$$

By combining Equations (S9), (S17), (S20), and (S28), we can find a similar equation for the temperature variation within the bubble:

$$\theta = (-\xi)\frac{R_0}{Jr}\left[C_1\left(1 + \frac{WB_1^2}{Pe^2D}\right)\sinh\left(B_1\frac{r}{R_0}\right) + C_2\left(1 + \frac{WB_2^2}{Pe^2D}\right)\sinh\left(B_2\frac{r}{R_0}\right)\right]. \tag{S30}$$

The coefficients C_1 and C_2, in Equations (S28) and (S30), can be found by considering the kinematic boundary condition:

$$u(r = R_0) = i\omega\xi R_0, \tag{S31}$$

and the temperature jump boundary condition, which we elaborate on below.

We assume that there is no mass flux across the liquid-gas interface, i.e. during evaporation or condensation. The diffusive lifetime of a nanobubble τ is given by:

$$\tau = \frac{R^2 M_g}{3D_c k_B T H}, \tag{S32}$$

where D_c is the diffusion coefficient, and H is Henry's law constant,[S3,S18] and Equation (S32) yields $\tau \sim 1\,\mu s$,[S3,S18] for a $R \sim 10\,nm$, compared to the typical oscillation periods used here $\sim$ 0.1–10 ns.[S6,S20,S21,S23] Given that the timescale for diffusive growth is much larger than the oscillation period of our nanobubbles, which is generally true for relatively insoluble gases, such as nitrogen used here,[S18] we can assume zero mass transfer across the liquid-gas

[3] The subscripts 1 and 2 refer to (+) and (−), respectively, in the (±) symbol in Equation (S29).

interface, i.e. the gas is non-condensable. See Section S2.1 below for further evidence to support this assumption from our MD simulations.

We also assume that the temperature variation in the *liquid* side is zero, which is justified when the liquid thermal conductivity K_l is much larger than the gas conductivity, i.e. $K_l \gg K$.[S21,S22] The temperature jump ΔT at a liquid-gas interface can then be found from the Smoluchowski boundary condition:

$$\Delta T = T(r = R) - T_0 = -\frac{2 - \alpha_T}{\alpha_T} \frac{2\kappa}{Pr\,(\kappa + 1)} Kn R_0 \frac{\partial T}{\partial r}\bigg|_{r=R_0}, \tag{S33}$$

where α_T is the thermal accommodation coefficient, Pr is the Prandtl number, given by $Pr = c_P \mu_g / K$, and Kn is the Knudsen number, given by $Kn = (\mu_g / R\rho)\sqrt{\pi M_g / (2k_B T)}$, or ratio between the mean free path and bubble radius.[S30,S31] While Equation (S35) is specifically derived for an ideal gas, we show this relation is suitable for predicting the temperature jumps in our MD simulations, in Section S4.

For simplicity, we group all the dimensionless numbers in the right hand side of Equation (S35) into a single coefficient β:

$$\beta = \frac{2 - \alpha_T}{\alpha_T} \frac{2\kappa}{Pr\,(\kappa + 1)} Kn, \tag{S34}$$

and expressing Equation (S35) in terms of dimensionless temperature and radial coordinate, we obtain the temperature jump boundary condition for the gas phase in our oscillating nanobubble:

$$\theta(r = R_0) = -\beta R_0 \frac{\partial \theta}{\partial r}\bigg|_{r=R_0}. \tag{S35}$$

C_1 and C_2 are found:

$$C_1 = \frac{Pe^2 D}{\sinh(B_1)} \frac{F_2}{F_2\lambda_1 - F_1\lambda_2}, \tag{S36a}$$

$$C_2 = \frac{Pe^2 D}{\sinh(B_2)} \frac{-F_1}{F_2\lambda_1 - F_1\lambda_2}, \tag{S36b}$$

where the expressions $\lambda_{1,2}$ and $F_{1,2}$ are given by:

$$\lambda_j = 1 - B_j \coth(B_j), \text{ for } j = 1, 2, \tag{S37}$$

and:

$$F_j = \left(Pe^2 D + W B_j^2\right)(1 - \beta \lambda_j), \text{ for } j = 1, 2, \tag{S38}$$

respectively.

The pressure at the bubble interface can then be expressed as $\phi(r = R_0) = (-\xi)\bar{\phi}(R_0)$, where $\bar{\phi}(R_0)$ reduces to:

$$\bar{\phi}(R_0) = Pe^2 D \frac{F_2 - F_1}{F_2 \lambda_1 - F_1 \lambda_2}. \tag{S39}$$

Equation (S8) can then be equated with Equation (S39) to determine the polytropic exponent k:

$$k = \frac{1}{3} \text{Real}\left\{\bar{\phi}(R_0)\right\}, \tag{S40}$$

and the thermal viscosity μ_{th}:

$$\mu_{th} = \frac{P_0 R_0^2}{\chi} \frac{\text{Imag}\left\{\bar{\phi}(R_0)\right\}}{4Pe}. \tag{S41}$$

In the MD simulations, k and μ_{th} are determined by fitting Eq. (S8) to the non-dimensional radius and pressure variations, and using Equation (S2) and extrapolating local pressure to the liquid-gas interface, as shown in Figure S1 for the $Pe = 8.5$ case. Since there is a velocity dependence on the bubble pressure (i.e. the radial and pressure oscillations are *not* in phase), the fitted line has a specific direction, as indicated by the arrows.

S2.1 Evidence for non-condensable gas in nanobubble

In our above derived nanobubble thermal oscillation model, we crucially assumed that no mass transfer occurred at the gas-liquid interface, which we justified by comparing the es-

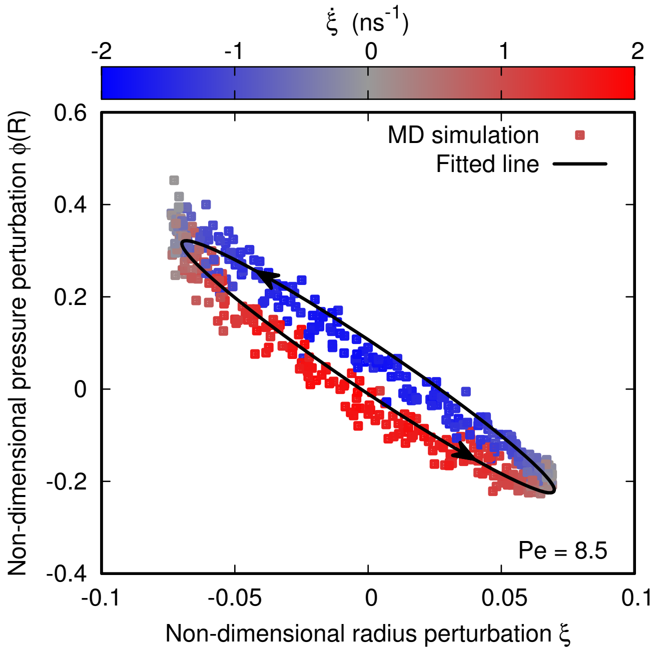

Figure S1: Variation in non-dimensional pressure $\phi(R)$ (measured at the liquid-gas interface of an oscillating nanobubble) with non-dimensional radius ξ, for the $Pe = 8.5$ case, from which the polytropic exponent k and thermal viscosity μ_{th} were fitted using Eq. (S8).

timated timescale for diffusive growth to the nanobubble's oscillation period. Here, we also show directly that there was negligible net mass transfer into the nanobubbles in our MD simulations.

In Figure S2, we count the number of N_2 and H_2O molecules within the fitted spherical profile of the nanobubble (see Section S1) in our MD simulations, for the $\omega = 2,\ 50\,\mathrm{rad/ns}$ cases. Also shown is the radius of the fitted spherical profile, showing the oscillation frequency of the system. Figure S2 clearly shows no significant change in bubble mass during oscillations, and no net change across the whole simulation ($< 1\%$ difference), and the contribution from vapor H_2O molecules is also negligible. Results in Figure S2 are averaged over several timesteps, so the number of molecules at each data point is not necessarily a whole number.

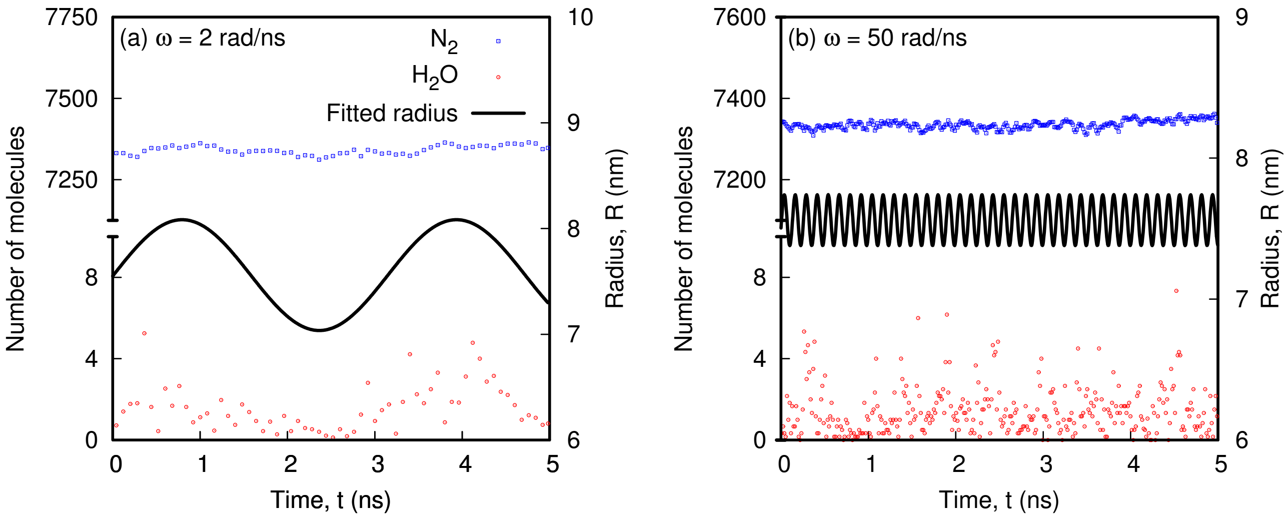

Figure S2: Variation in number of N_2 and H_2O molecules in the nanobubble, and radius of the nanobubble's fitted spherical profile during oscillations, for the (a) $\omega = 2\,\mathrm{rad/ns}$ and (b) $\omega = 50\,\mathrm{rad/ns}$ cases. Note the split in the left y axis.

S2.2 Pressure and temperature variations in nanobubble

Along with Figure 3 in the main text, we plot the radial pressure and temperature variations, in Figures S3 and S4, respectively, for various other cases of Pe. See Table 1 in the main article, for the description of the different models used. In each case, our NB2 model, assuming non-ideal gas and non-equilibrium temperature jump at the liquid-gas interface, gives best agreement with the MD simulations. Figure S3(a)–(d) shows the gradual transition to compressible gas behavior, as pressure becomes more non-uniform across the nanobubble, with increasing Pe.

S3 Van der Waals equation of state

We ran many equilibrium MD NVT ensemble simulations using the two-site N_2 model, to determine the vdW equation of state parameters in Equation (S4). Simulations were run in a periodic $482\,\mathrm{nm}^3$ cube, with fixed temperatures ranging from 240–370 K using a Nosé–Hoover thermostat, and densities varying between 60–210 $\mathrm{kg/m}^3$. All results in density, temperature,

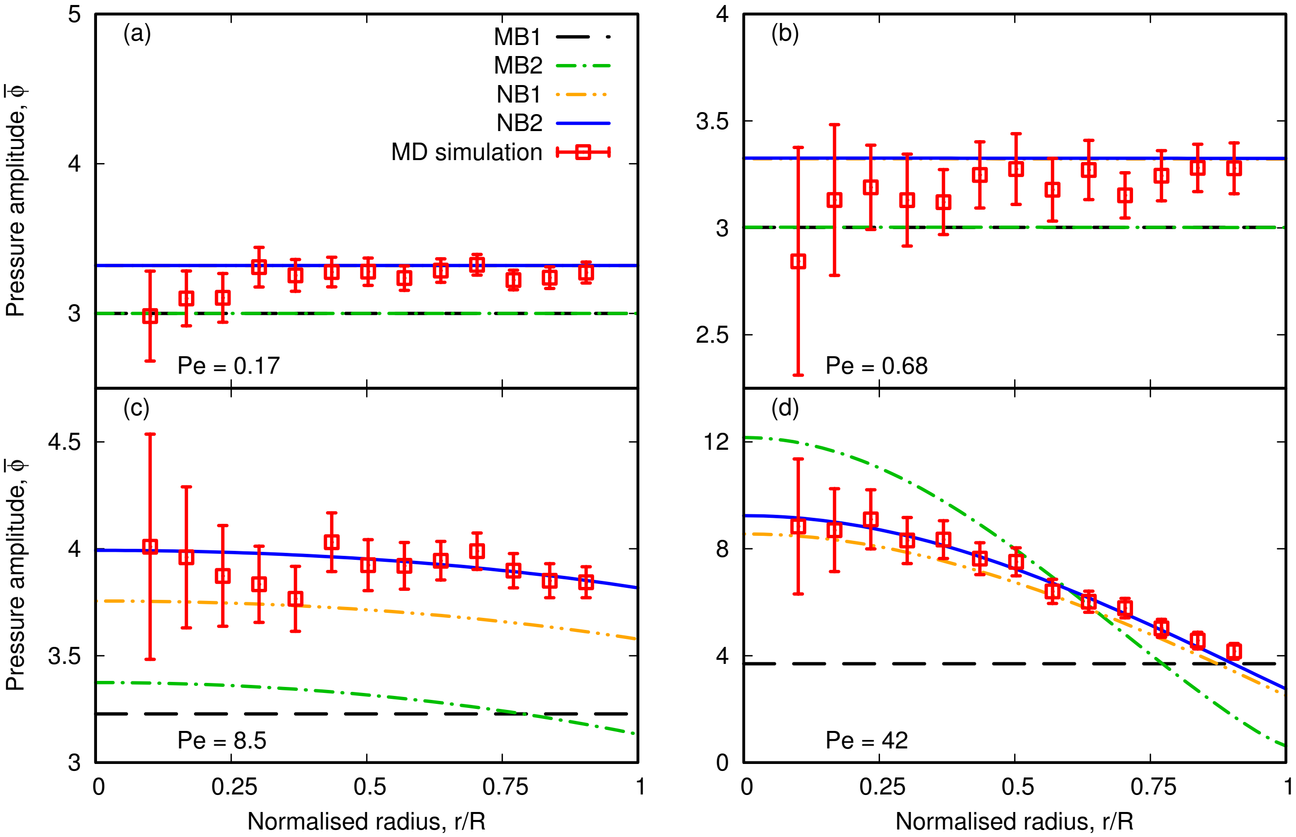

Figure S3: Radial variations of pressure $\bar{\phi}$ amplitudes in the oscillating nanobubbles, for the: (a) $Pe = 0.17$, (b) $Pe = 0.68$, (c) $Pe = 8.5$, and (d) $Pe = 42$ cases.

and pressure were averaged over 10 ns at equilibrium, and are shown in Figure S5.

We first compare our MD results with the ideal gas equation of state $P = \rho k_B T/M_g$, in Figure S5(a). The residuals δP are calculated as the difference between the measured pressure in the MD simulations, and the expected pressure from the equation of state, at a given density and temperature, with the root-mean-square of the residuals for the ideal gas equation of state found to be $\delta P_{rms} = 0.628\,\text{MPa}$. Figure S5(b) shows the MD simulation results, compared with the vdW equation of state, using least squares fitted parameters: $A = (156.1 \pm 0.9)\text{J}\,\text{m}^3/\text{kg}^2$ and $B = (1.446 \pm 0.005) \times 10^{-3}\,\text{m}^3/\text{kg}$. The vdW equation of state provides a much better fit to the MD simulation data, than the ideal gas law, with $\delta P_{rms} = 0.0559\,\text{MPa}$.

We fitted the vdW coefficients for densities, pressures, and temperatures near our region of interest for our nanobubble gas phase, although these coefficients could also be estimated

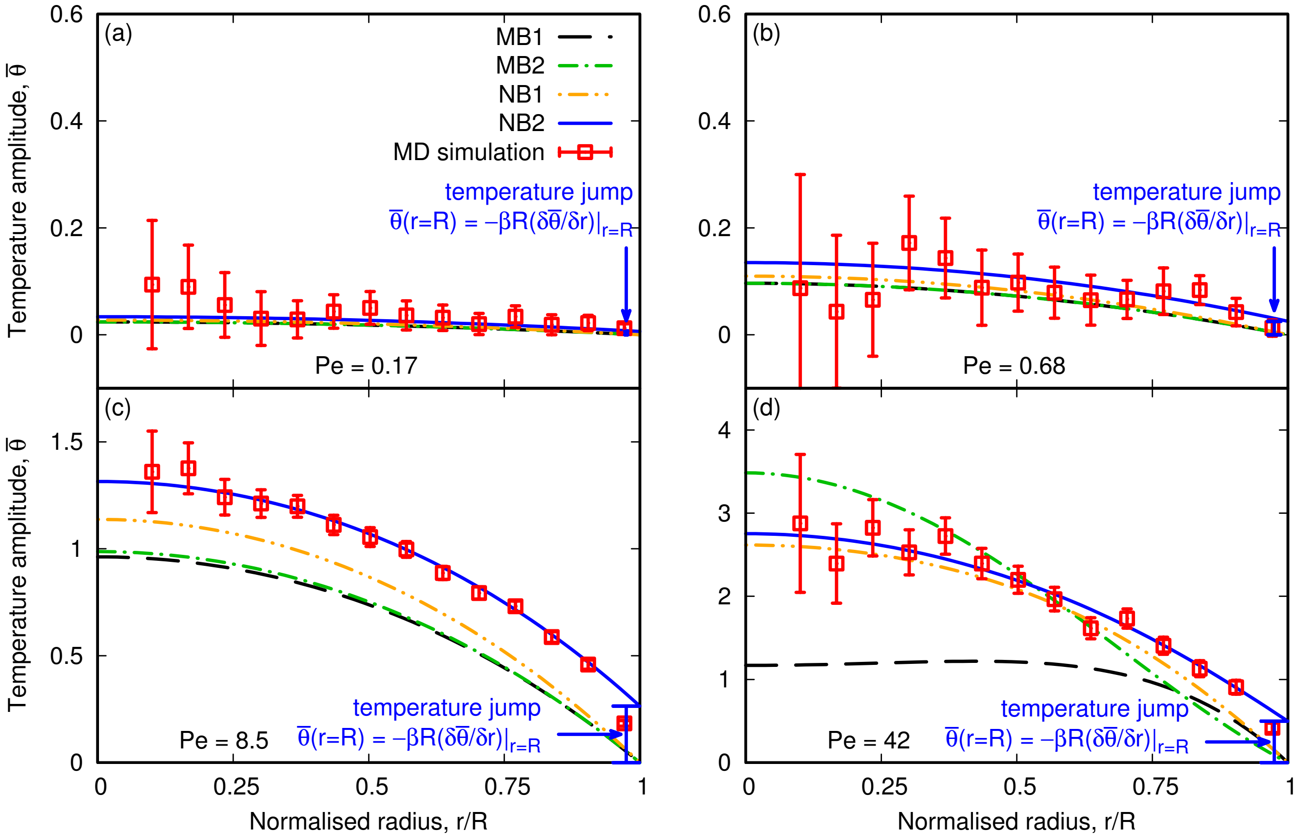

Figure S4: Radial variations of temperature $\bar{\theta}$ amplitudes in the oscillating nanobubbles, for the: (a) $Pe = 0.17$, (b) $Pe = 0.68$, (c) $Pe = 8.5$, and (d) $Pe = 42$ cases.

from the critical point of fluid:

$$A = \frac{27k_B^2 T_c^2}{64M_g^2 P_c}, \tag{S42}$$

and

$$B = \frac{k_B T_c}{8M_g P_c}, \tag{S43}$$

where T_c and P_c are the critical temperature and pressure, respectively.[S17] Using experimentally measured values for the critical temperature and pressure of nitrogen: $T_c = 126.2\,\mathrm{K}$ and $P_c = 3.400\,\mathrm{MPa}$, respectively, we obtain vdW parameters $A = 174.08\,\mathrm{J\,m^3/kg^2}$ and $B = 1.3771 \times 10^{-3}\,\mathrm{m^3/kg}$, from Equations (S42) and (S43), respectively, which we compared to our MD results in Figure S6.

Note, there are some differences between our obtained parameters when fitting to our MD simulation data, and the parameters obtained from the critical point, for various reasons.

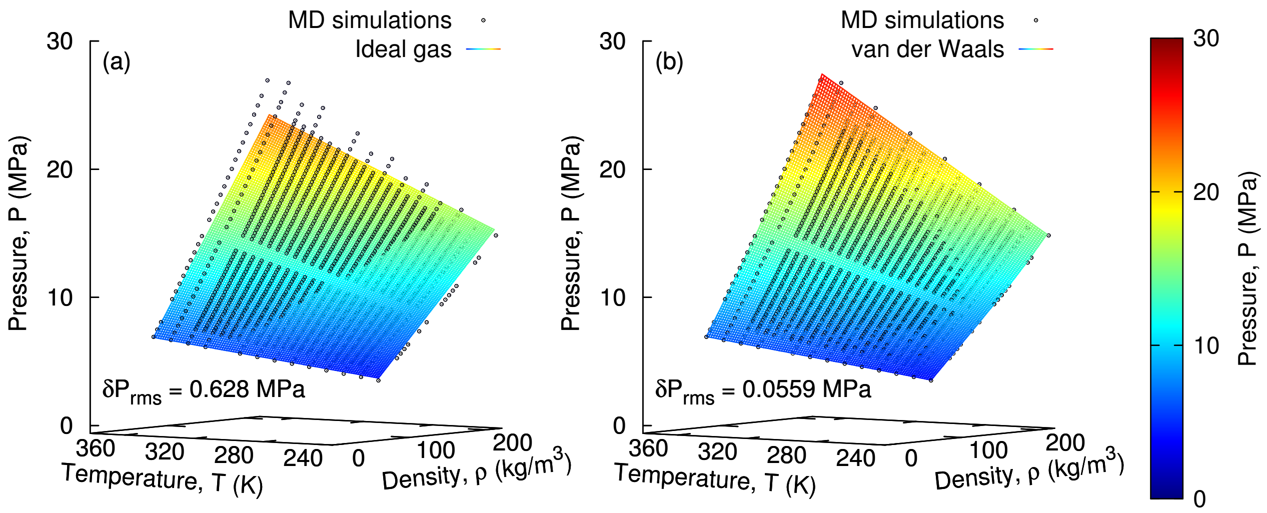

Figure S5: Variation in density, temperature and pressure for equilibrium NVT MD simulations of two-site N_2 model. Results are compared to: (a) the ideal gas law $P = \rho k_B T/M_g$; and (b) vdW equation of state, as given in Equation (S4), using obtained parameters from least-squares fitting. The root-mean-square of the residuals δP_{rms} are also given for each equation of state.

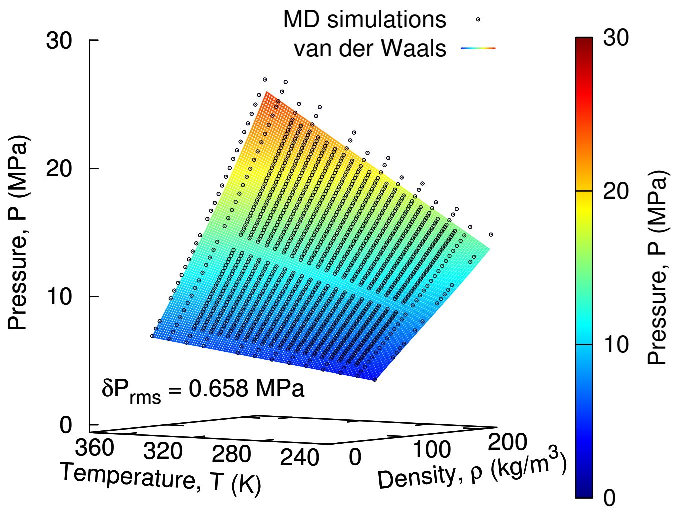

Figure S6: Variation in density, temperature and pressure for equilibrium NVT MD simulations of two-site N_2 model, with results compared to the vdW equation of state in Equation (S4), using parameters obtained from Equations (S42) and (S43). The root-mean-square of the residuals δP_{rms} are also given.

First, we employ a cut-off distance in our MD simulations (see Section S1), in order to reduce computational cost, and we do not employ long-range tail correction solvers, as these are not suitable for inhomogenous and multiphase systems, such as in our nanobubble simulations.

Introducing these cut-off radii can significantly alter the resulting critical point of the LJ fluid by orders of 10 %,[S32] while Monte Carlo simulations of similar two-site N_2 models (without a cut-off) provide reasonably good agreement with nitrogen's critical point.[S33,S34]

Second, our interest in using the vdW equation of state is to obtain linearized variables W and J at conditions similar to the interior of our nanobubble to obtain best agreement with our MD simulations, hence, we employed our data fitting to this region in particular, instead of near the critical point. Our model derived in Section S2 is general enough that other equations of state, such as Redlich–Kwong[S35] or Benedict–Webb–Rubin,[S36] could alternatively be used to obtain expressions for W and J. Using the critical point to estimate vdW parameters provides suitable estimates for a broad range of nanobubble sizes, as we have done in Figure 5 in the main article.

S4 Molecular Dynamics simulations of Knudsen temperature jump

We ran Non-Equilibrium Molecular Dynamics (NEMD) heat source and sink simulations, using the same two-site nitrogen (N_2), TIP4P/2005 water (H_2O) and wall (W) models, as described in Section S1, to accurately measure the temperature jump across the liquid-gas interface. The liquid, gas, and wall phases were arranged in a slab formation, as shown in Figure S7(a), with full periodic boundary conditions. The gas phase was initialized with a width of 15 nm, and density 159 kg/m^3, i.e. identical to the nanobubble diameter ($2R_0$) and measured internal density in Section S1, respectively. We equilibrated the surrounding liquid to obtain diffusive equilibrium with the high internal gas pressure.[S4] A Nosé–Hoover thermostat was used to maintain the wall atoms at 300 K, while a central region at $x = 10.6$–12.6 nm was thermostated at a temperature varying between 250–350 K (depending on the simulation case), using a Berendsen thermostat, to establish a temperature gradient in the gas. All other atoms were run without a thermostat.

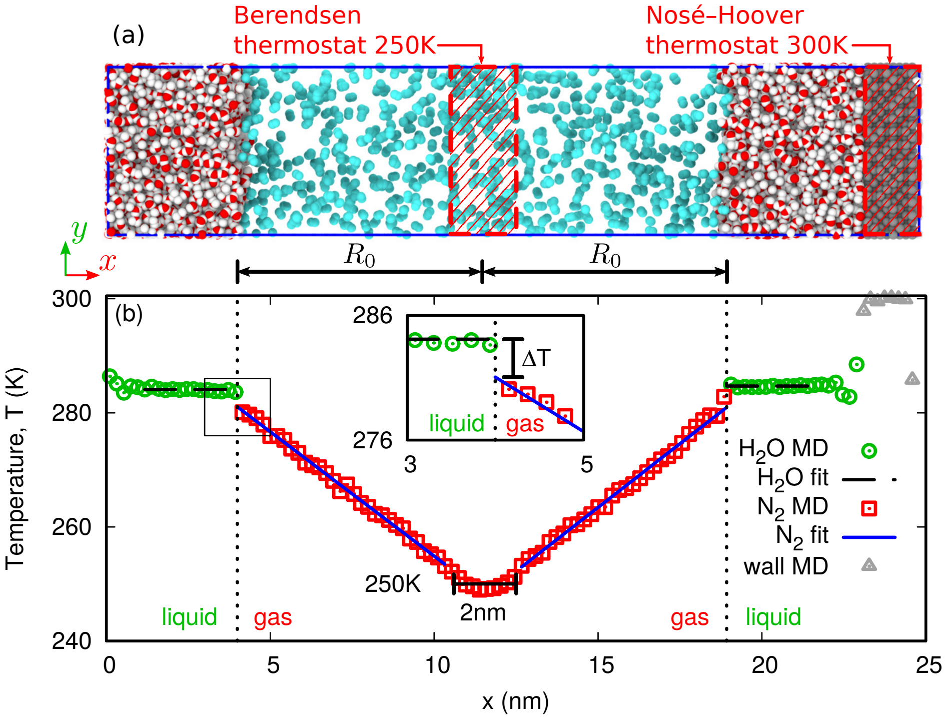

Figure S7: (a) MD simulation setup for investigating the temperature jump at the liquid-gas interface; atoms have the same colour scheme as in Figure 1(d) in the main article. (b) Variation in temperature across the fluid, for the 250 K gas temperature case. The dotted lines show the x-coordinates of the liquid-gas interfaces, found from the 50 % isodensity contours. The inset in (b) shows the temperature jump ΔT at the liquid-gas interface in more detail.

Temperature across the system was measured locally in bins, and is plotted in Figure S7(b), for the 250 K gas temperature case. Straight lines were fitted to the gas phase temperature profiles in the bulk region (outside the central thermostated region) and 0.5 nm away from the liquid-gas interfaces (found from the 50 % isodensity contours), using least squares fitting, while a horizontal mean line was fitted to the liquid phase temperatures. We define the temperature jump ΔT as the difference in fitted temperature profiles in each fluid, extrapolated to the liquid-gas interface, as shown in more detail inset in Figure S7(b). We also observe significant temperature jumps ~ 10 K at the liquid-wall interfaces, resulting from the Kapitza resistance,[S37] however, this has no effect on the liquid-gas temperature jump, which is the focus of our work.

The liquid-gas temperature jump is plotted as a function of the fitted temperature gra-

dients $\partial T/\partial x$ in Figure S8. We fit a straight line to the data in Figure S8 (shown as a

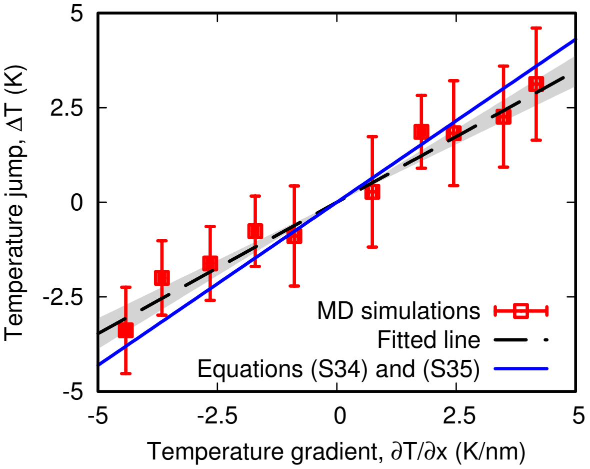

Figure S8: Variation in temperature jump ΔT at the liquid-gas interface, with the temperature gradient in the gas phase $\partial T/\partial x$. The gradients of the straight lines are equal to βR_0, with the black dashed line fitted to the MD data (the grey region shows the error bounds), and solid blue line found from Equations (S34) and (S35).

black dashed line), which has a gradient equal to βR_0, where $\beta = 0.09(\pm 0.01)$ (error bounds shown as grey region in Figure S8), and is in good agreement with the value $\beta = 0.12$, found from the analytical expression in Equation (S34), when taking a thermal accommodation coefficient $\alpha_T = 1$.

S5 Van der Waals polytropic gas law limits

In this section, we derive the revised limits for isothermal and adiabatic expansion for a vdW gas. First, we rewrite the polytropic gas law (Equation (S7), neglecting the thermal viscosity μ_{th}) as:

$$\frac{P}{P_0} = \left(\frac{\rho}{\rho_0}\right)^k. \tag{S44}$$

Linearizing Equation (S44) for small perturbations in ϕ and η yields:

$$\phi = k\eta. \tag{S45}$$

For isothermal expansion, we can simply equate Equation (S45) with (S9) when $\theta = 0$, to find $k = W$.

For adiabatic (isentropic) expansion, we use the relation:[S38]

$$k = \frac{c_P}{c_V}\frac{\rho_0}{P_0}\left(\frac{\partial P}{\partial \rho}\right)_T, \tag{S46}$$

and recalling our earlier definitions: $\kappa = c_P/c_V$ and $W = (\rho_0/P_0)(\partial P/\partial \rho)_T$, we find $k = \kappa W$ during adiabatic expansion.

References

(S1) Plimpton, S. Fast Parallel Algorithms for Short-Range Molecular Dynamics. *J. Comput. Phys.* **1995**, *117*, 1–19.

(S2) Dockar, D.; Borg, M. K.; Reese, J. M. Mechanical Stability of Surface Nanobubbles. *Langmuir* **2019**, *35*, 9325–9333.

(S3) Epstein, P. S.; Plesset, M. S. On the Stability of Gas Bubbles in Liquid-Gas Solutions. *J. Chem. Phys.* **1950**, *18*, 1505–1509.

(S4) Zambrano, H. A.; Walther, J. H.; Jaffe, R. L. Molecular Dynamics Simulations of Water on a Hydrophilic Silica Surface at High Air Pressures. *J. Mol. Liq.* **2014**, *198*, 107–113.

(S5) Abascal, J. L. F.; Vega, C. A General Purpose Model for the Condensed Phases of Water: TIP4P/2005. *J. Chem. Phys.* **2005**, *123*, 234505.

(S6) Dockar, D.; Gibelli, L.; Borg, M. K. Forced oscillation dynamics of surface nanobubbles. *J. Chem. Phys.* **2020**, *153*, 184705.

(S7) Dockar, D.; Gibelli, L.; Borg, M. K. Shock-induced collapse of surface nanobubbles. *Soft Matter* **2021**, *17*, 6884–6898.

(S8) J. H. Irving and J. G. Kirkwood, The Statistical Mechanical Theory of Transport Processes. IV. The Equations of Hydrodynamics. *J. Chem. Phys.* **1950**, *18*, 817–829.

(S9) Thompson, A. P.; Plimpton, S. J.; Mattson, W. General formulation of pressure and stress tensor for arbitrary many-body interaction potentials under periodic boundary conditions. *J. Chem. Phys.* **2009**, *131*, 154107.

(S10) Lemmon, E. W.; McLinden, M. O.; Friend, D. G. *NIST Chemistry WebBook, NIST Standard Reference Database Number 69, Eds. P.J. Linstrom and W.G. Mallard*; National Institute of Standards and Technology: Gaithersburg, 20899, 2017; Chapter Thermophysical properties of fluid systems.

(S11) Janeček, J. Long Range Corrections in Inhomogeneous Simulations. *J. Phys. Chem. B* **2006**, *110*, 6264–6269, PMID: 16553443.

(S12) Matsumoto, M.; Kataoka, Y. Study on liquid–vapor interface of water. I. Simulational results of thermodynamic properties and orientational structure. *J. Chem. Phys.* **1988**, *88*, 3233–3245.

(S13) Shi, B.; Sinha, S.; Dhir, V. K. Molecular dynamics simulation of the density and surface tension of water by particle-particle particle-mesh method. *J. Chem. Phys.* **2006**, *124*, 204715.

(S14) Pillai, R.; Borg, M. K.; Reese, J. M. Acoustothermal Atomization of Water Nanofilms. *Phys. Rev. Lett.* **2018**, *121*, 104502.

(S15) Datta, S.; Pillai, R.; Borg, M. K.; Sefiane, K. Acoustothermal Nucleation of Surface Nanobubbles. *Nano Lett.* **2021**, *21*, 1267–1273, PMID: 33494609.

(S16) Çengel, Y. A.; Boles, M. A. *Thermodynamics: An Engineering Approach*, 7th ed.; McGraw–Hill, 2011.

(S17) Johnston, D. C. *Advances in Thermodynamics of the van der Waals Fluid*; 2053-2571; Morgan & Claypool Publishers, 2014.

(S18) Lohse, D.; Zhang, X. Surface Nanobubbles and Nanodroplets. *Rev. Mod. Phys.* **2015**, *87*, 981–1035.

(S19) Brotchie, A.; Zhang, X. H. Response of interfacial nanobubbles to ultrasound irradiation. *Soft Matter* **2011**, *7*, 265–269.

(S20) Brennen, C. E. *Cavitation and Bubble Dynamics*; Cambridge University Press, 2013.

(S21) Prosperetti, A. Thermal effects and damping mechanisms in the forced radial oscillations of gas bubbles in liquids. *J. Acoust. Soc. Am.* **1977**, *61*, 17–27.

(S22) Prosperetti, A.; Crum, L. A.; Commander, K. W. Nonlinear bubble dynamics. *J. Acoust. Soc. Am.* **1988**, *83*, 502–514.

(S23) Prosperetti, A. The thermal behaviour of oscillating gas bubbles. *J. Fluid Mech.* **1991**, *222*, 587–616.

(S24) Devin, C. Survey of Thermal, Radiation, and Viscous Damping of Pulsating Air Bubbles in Water. *J. Acoust. Soc. Am.* **1959**, *31*, 1654–1667.

(S25) Rayleigh, L. VIII. On the pressure developed in a liquid during the collapse of a spherical cavity. *London, Edinburgh Dublin Philos. Mag. J. Sci.* **1917**, *34*, 94–98.

(S26) Plesset, M. S. The dynamics of cavitation bubbles. *J. Appl. Mech.* **1949**, *16*, 277–282.

(S27) Eucken, A. Über das Wärmeleitvermögen, die spezifische Wärme und die innere Reibung der Gase. *Phys. Z.* **1913**, *14*, 324–332.

(S28) Chapman, D.; Cowling, T. G. *The Mathematical Theory of Non-Uniform Gases*, 3rd ed.; Cambridge University Press, 1970.

(S29) Kennard, E. H. *Kinetic Theory of Gases*, 1st ed.; McGraw–Hill, 1938.

(S30) Su, W.; Li, Q.; Zhang, Y.; Wu, L. Temperature jump and Knudsen layer in rarefied molecular gas. *Phys. Fluids* **2022**, *34*, 032010.

(S31) Lockerby, D. A.; Reese, J. M. High-resolution Burnett simulations of micro Couette flow and heat transfer. *J. Comput. Phys.* **2003**, *188*, 333–347.

(S32) Smit, B. Phase diagrams of Lennard-Jones fluids. *J. Chem. Phys.* **1992**, *96*, 8639–8640.

(S33) Galassi, G.; Tildesley, D. J. Phase Diagrams of Diatomic Molecules Using the Gibbs Ensemble Monte Carlo Method. *Mol. Simul.* **1994**, *13*, 11–24.

(S34) Eskandari Nasrabad, A.; Laghaei, R. Thermodynamic and transport properties of nitrogen fluid: Molecular theory and computer simulations. *Chem. Phys.* **2018**, *506*, 36–44.

(S35) Redlich, O.; Kwong, J. N. S. On the Thermodynamics of Solutions. V. An Equation of State. Fugacities of Gaseous Solutions. *Chem. Rev.* **1949**, *44*, 233–244, PMID: 18125401.

(S36) Benedict, M.; Webb, G. B.; Rubin, L. C. An Empirical Equation for Thermodynamic Properties of Light Hydrocarbons and Their Mixtures II. Mixtures of Methane, Ethane, Propane, and n-Butane. *J. Chem. Phys.* **1942**, *10*, 747–758.

(S37) Alosious, S.; Kannam, S. K.; Sathian, S. P.; Todd, B. D. Prediction of Kapitza resistance at fluid-solid interfaces. *J. Chem. Phys.* **2019**, *151*, 194502.

(S38) Kouremenos, D. A.; Kakatsios, X. K. The three isentropic exponents of dry steam. *Forsch. Ingenieurwes.* **1985**, *51*, 117–122.
